# *Lactococcus* *G423* Improves Lipid Metabolism in Broilers Through the Gut Microbiota and AMPK Signaling Pathway

**DOI:** 10.3390/microorganisms14092008

**Published:** 2026-09-10

**Authors:** Bo Pang, Ruixiang Li, Xinyu Wang, Weitong Guan, Wei Ma, Chunqiang Wang, Desheng Li, Mi Wang

**Affiliations:** 1 College of Animal Husbandry and Veterinary Medicine, Jinzhou Medical University, Jinzhou 121010, China; 2 Collaborative Innovation Center for Prevention and Control of Zoonoses, Jinzhou Medical University, Jinzhou 121010, China

**Keywords:** *Lactococcus*, broilers, AMPK, PPAR, lipid metabolism

## Abstract

This study aimed to elucidate the role of *Lactococcus* G423 in regulating the AMPK (AMP-activated protein kinase) signaling pathway and gut microbiota, and to investigate the mechanisms underlying its potential to improve lipid metabolism in broilers. In this trial, 960 one-day-old Arbor Acres (AA) broilers were randomly assigned to one of three groups: a control group (CON), a low-dose *Lactococcus* G423 group (Lac-L), and a high-dose *Lactococcus* G423 group (Lac-H). Each group consisted of eight replicates, with 40 individuals per replicate. Compared with the CON group, the Lac-L and Lac-H groups showed no significant differences in ADFI (Average Daily Feed Intake), ADG (Average Daily Gain), FCR (Feed Conversion Ratio), breast muscle yield, leg muscle yield, or abdominal fat yield (*p* > 0.05). Compared with the control group, neither Lac-L nor Lac-H affected serum TG (Triglyceride) or LDL (Low-Density Lipoprotein) levels (*p* > 0.05). However, both Lac-L and Lac-H significantly decreased serum CHO (Cholesterol) and increased serum HDL (High-Density Lipoprotein) (*p* < 0.05). Regarding the AMPK signaling pathway, both the Lac-L and Lac-H groups significantly downregulated the hepatic mRNA expression of Acetyl-CoA carboxylase (ACC) (*p* < 0.05). The Lac-L groups significantly increased the mRNA expression of AMP-activated protein kinase (AMPK) and Sterol Regulatory Element-Binding Protein 1 (SREBP1) (*p* < 0.05), and the Lac-H groups significantly increased the mRNA expression of Apolipoprotein A1 (APOA1) and Protein kinase B (PKB) (*p* < 0.05) and significantly reduced the mRNA expression of Fatty acid-binding protein 1 (FABP1) (*p* < 0.05), compared to the CON group. Gut microbiota analysis indicated that both treatment groups exhibited significantly increased α-diversity indices (*p* < 0.05) and altered microbial composition at an order level comparable with that of the CON group. Specifically, the abundances of beneficial families such as *Lachnospirales* and *Lactobacillales* were elevated, while significant changes were observed in other taxa including *Ruminococcaceae* and *Akkermansiaceae* (*p* < 0.05). These changes corresponded with significant differences in the MDI and GMHI (*p* < 0.05). In conclusion, *Lactococcus* G423 may regulate lipid metabolism in broilers through the combined modulation of the AMPK signaling pathway and the gut microbiota. Specifically, *Lactococcus* G423 appears to regulate broiler lipid metabolism by modulating the gut microbiota–hepatic lipid metabolism axis.

## 1. Introduction

Modern livestock production aims to maximize profitability, often through selecting for excessively high growth rates in broiler chickens. In recent decades, the genetic selection for body weight, growth rate, and feed conversion rate of broiler chickens has contributed to a prominent increase in production efficiency; however, excessive deposition of body fat has increased. The drawback has severely compromised the economic benefits in the poultry industry. Consequently, strategies to reduce lipid deposition have become a major research focus.

The AMPK signaling pathway is a central regulator of lipid metabolism [1]. AMPK signaling is modulated by the cellular AMP/ATP ratio and by the expression of its upstream kinase, LKB1 [2,3]. Activation of AMPK enhances systemic glucose uptake, promotes fatty acid oxidation in the liver, and reduces lipogenesis [4]. Specifically, AMPK activation downregulates the expression of key lipogenic genes such as SREBP1, ACC, and HMGCR, thereby inhibiting fatty acid and cholesterol synthesis [5,6,7]. Conversely, it upregulates genes involved in fatty acid oxidation and lipid catabolism, including CPT1 and PGC1α (Peroxisome proliferator-activated receptor gamma coactivator-1 alpha) to promote these processes [8,9]. AMPK also interacts with PPAR to coordinately regulate lipid metabolism [10]. As a downstream signaling component of AMPK, PPAR activity is modulated by AMPK. Activation of AMPK inhibits the activity of PPAR, SREBP1, and ACC, which collectively suppresses adipogenesis and lipid synthesis [11,12].

The gut microbiome is a highly complex and diverse ecosystem that harbors both beneficial probiotics and potential pathogens [13]. Notably, supplementing with probiotics can actively reshape the composition of the intestinal microbial community [14,15]. Probiotics are highly regarded for their potent anti-inflammatory effects, capacity to enhance host antioxidant status, improve growth performance, and positively modulate host nutritional metabolism [16,17].

Shifts in gut microbial composition are closely linked to host lipid metabolism. In broilers, comparative studies on gut microbiota between high- and low-abdominal-fat deposition groups have revealed distinct microbial signatures. For instance, a higher relative abundance of Sphaerochaeta is observed in the low-fat group, along with a significantly elevated serum high-density lipoprotein (HDL) concentration [18]. Furthermore, an increased *Bacteroides*-to-*Firmicutes* ratio, as well as taxa such as *Mucispirillum*, are associated with reduced fat deposition. Some *Actinobacteria* may alleviate obesity through the production of secondary metabolites [19]. For example, *Parabacteroides distasonis* can increase intestinal succinate levels and alter the distribution of secondary bile acids, thereby affecting lipid metabolism [20].

In the livestock sector, *lactic acid bacteria* (LAB) are the most widely utilized probiotic microorganisms [21,22]. Relevant studies have shown that *Lactococcus lactis* can enhance the growth performance of broilers and promote intestinal development [23,24]. Research suggests that LAB can regulate energy metabolism in mice by modifying the gut microflora, thereby influencing fat accumulation [25]. In murine models, LAB supplementation has been shown to increase final body weight and modulate obesity induced by a high-fat diet. This is achieved by elevating serum HDL, reducing total cholesterol (TCHO) and low-density lipoprotein (LDL) levels, and altering adipocyte size [26]. In broilers under heat stress, dietary supplementation with lactic acid bacteria significantly increases final body weight, average daily gain, and average daily feed intake, while reducing the feed-to-gain ratio. It also significantly increases intramuscular fat (IMF) content, by regulating lipid metabolism [27]. Although our previous research found that adding *Lactococcus G423* can affect the gut microbiota and influence lipid metabolism, whether it can further affect liver gene signaling pathways has not yet been confirmed [28]. Therefore, in this experiment, *Lactococcus* G423 is studied for its effect on lipid metabolism. The proposed mechanism involves the modulation of gut microbiota, which then influences the hepatic AMPK signaling pathway via the gut–liver axis, leading to improved lipid metabolism.

## 2. Materials and Methods

### 2.1. Birds, Diets, and Experimental Design

A total of 960 one-day-old Arbor Acres (AA) broilers were randomly assigned to three experimental groups. Each treatment consisted of 320 birds (eight replicates of 40 birds). The feeding trial was divided into two phases: the starter phase (days 1–21) and the grower phase (days 22–42). A basal diet was formulated to meet the nutritional requirements recommended by the Chinese Broiler Feeding Standards (NY/T33-2004) [29] (Table 1). Three dietary groups were provided: (1) a control group, which were fed the basal diet; (2) a low-dose *Lactococcus* group (Lac-L), which were fed the basal diet supplemented with 50 g/kg of *Lactococcus*; and (3) a high-dose *Lactococcus* group (Lac-H), which were fed the basal diet supplemented with 100 g/kg of *Lactococcus*. *Lactococcus G423* (1 × 10^10^ CFU/g) were mixed in the basal diet. The freeze-dried powder of *Lactococcus G423* used in this study was purchased from Tianjin Ding Zheng Xin Xing Biotechnology Co., Ltd. (Tianjin, China). Product specifications: *Lactococcus G423* ≥ 1 × 10^10^ CFU/g. Culture condition: M17 (Qingdao Hope Bio-Technology Co., Ltd., Wuhan, China) + 0.5% glucose, 30°.

### 2.2. Bird Management

Broiler chickens were raised in cages. Prior to the experiment, chicken houses, waterers, and feed troughs were thoroughly cleaned and disinfected, followed by window ventilation. The environmental temperature in the housing was maintained at 33–35 °C with humidity between 60% and 70%. Temperature was gradually reduced by 2–3 °C per week until it reached 22–23 °C when the birds were 4 weeks of age, after which it remained constant. A total of 960 broilers were included in this study. All birds were observed daily at fixed times for feeding, drinking, behavior, and fecal conditions. Sick or weak birds were promptly isolated for treatment or culled to prevent disease spread.

### 2.3. Growth and Carcass Measurements

Broiler performance was evaluated weekly in terms of ADG, ADFI, and FCR. The ADG, ADFI, and FCR were calculated and are presented for the entire 6-week experimental period. On day 42, 16 birds per treatment group with similar body weights and an equal sex ratio (half male and half female) were selected for carcass trait analysis. Prior to slaughter, birds were fasted for 12 h and individually weighed. The percentages of carcass yield, eviscerated yield, semi-eviscerated yield, breast muscle, thigh muscle, and abdominal fat were calculated, relative to live body weight. The liver was collected and immediately sampled into a 2 mL cryogenic tube, then immediately placed in liquid nitrogen for temporary storage before being stored in a −80 °C freezer for long-term preservation, in order to facilitate subsequent PCR testing. The cecum was collected, and the cecal microbiota was scraped into a 1.5 mL centrifuge tube and then temporarily stored in liquid nitrogen. After collection, this was immediately sent for testing, to conduct gut microbiota analysis.

### 2.4. Enzyme-Linked Immunosorbent Assay

Blood samples (10 mL) were collected from the jugular vein from birds in each group, and immediately placed on ice. Serum was obtained by centrifugation at 4 °C and 3000× *g* for 15 min and stored at −20 °C until analysis. Serum concentrations of HDL, LDL, TG, and CHO were determined using an enzyme-labeled instrument (Hangzhou Aosheng Instrument Co., Ltd., Hangzhou, China), according to the instructions of the commercial enzymatic colorimetric assays. (Nanjing Jiancheng Bioengineering Institute, Nanjing, Jiangsu, China; Catalog numbers: A113-1-1; A112-1-1; A111-1-1; A110-1-1).

### 2.5. Illumina MiSeq Sequencing for Intestinal Microbial Diversity Analysis

Eight cecal content samples per group were randomly selected for intestinal microbiota analysis. Eight samples of cecal contents were randomly collected from each group, placed in 2 mL cryogenic tubes, immediately preserved in liquid nitrogen, and subsequently used for gut microbiota analysis. The hypervariable V3-V4 region of the bacterial 16S rRNA gene was amplified by PCR using the universal primer set 338F (5′-ACTCCTACGGGAGGCAGCAG-3′) and 806R (5′-GGACTACHVGGGTWTCTAAT-3′). DNA quality and concentration were assessed by 1.0% agarose gel electrophoresis and a NanoDrop^®^ ND-2000 spectrophotometer (Thermo Scientific Inc., Waltham, MA, USA), after which samples were stored at −80 °C until further use. All samples were amplified in triplicate. PCR products were extracted from a 2% agarose gel and purified with the AxyPrep DNA Gel Extraction Kit (Axygen Biosciences, Union City, CA, USA), following the manufacturer’s instructions. DNA concentration was then quantified using a Quantus™ Fluorometer (Promega, Madison, WI, USA). Paired-end sequencing (2 × 300 bp) was performed on an Illumina MiSeq platform (Illumina, San Diego, CA, USA). Raw sequence reads were quality-filtered and merged using FLASH (V1.2.8), followed by open-reference operational taxonomic unit (OTU) clustering via UPARSE and taxonomic classification against the SILVA 16S rRNA database (Release 138). Alpha diversity was assessed via Shannon, Simpson, Ace, Sobs and Chao1 indices, and beta diversity was evaluated through principal coordinates analysis (PCoA) based on Bray–Curtis dissimilarity matrices. Non-parametric Kruskal–Wallis test and linear discriminant-analysis effect size (LefSe) using linear discriminant analysis (LDA) scores were used to evaluate differential bacteria. The threshold of LDA scores was 3.0. Microbial Dysbiosis Index (MDI) and Gut Microbiome Health Index (GMHI) were analyzed using Python (version 2.7.10).

### 2.6. Quantitative PCR (qPCR)

Liver tissue samples were individually homogenized, and total RNA was extracted using TRIzol^®^ reagent. First-strand cDNA was synthesized from total RNA using the Superscript First-Strand Synthesis System (Life Technologies, Grand Island, NY, USA). During slaughter, 8 liver samples were randomly selected from each group, placed into 2 mL cryovials, immediately put into liquid nitrogen, and finally transferred to a −80 °C freezer for storage. The abundance of target transcripts was quantified by RT-qPCR (Life Technologies, Grand Island, NY, USA). β-Actin was used as an internal control in this study. The gene-specific primers, the size of the product, and GenBank accession are shown in Table 2.

### 2.7. Statistical Analysis

Between-group statistical differences were compared using one-way analysis of variance (ANOVA), followed by post hoc multiple comparisons using Fisher’s least significant difference (LSD) *t*-test. Data analyses were performed using SPSS 22.0 statistical software. Results are presented as mean ± standard error of the mean (SEM). Differences were considered statistically significant at *p* < 0.05. For qPCR data, ΔCt was calculated as (mean Ct of the target gene)—(mean Ct of the internal reference gene). ΔΔCt was then derived as ΔCt—(mean ΔCt of the control group).

Microbial 16S rRNA gene sequencing data were analyzed using an online platform (https://cloud.majorbio.com).

## 3. Results

### 3.1. Growth and Carcass Performance

Compared with the control group, ADG, ADFI and FCR, there were no significant changes in the Lac-H and Lac-L groups in 42 days (*p* > 0.05). The dressing percentage, eviscerated weight, semi-eviscerated weight, and breast- and thigh-muscle yields of AA broilers showed no significant changes (*p* > 0.05) (Table 3). However, the abdominal fat percentage was reduced by supplementation with Lac-L and Lac-H, although these reductions were not statistically significant (*p* > 0.05) (Table 3).

### 3.2. Serum Biochemical Parameters

Compared with the control group, Lac-L and Lac-H groups significantly decreased the serum CHO and increased the serum HDL level (*p* < 0.05). However, neither Lac-L nor Lac-H affected the serum TG or LDL levels (*p* > 0.05) (Table 4).

### 3.3. AMPK Signaling Pathway Analysis

**Figure 1:**
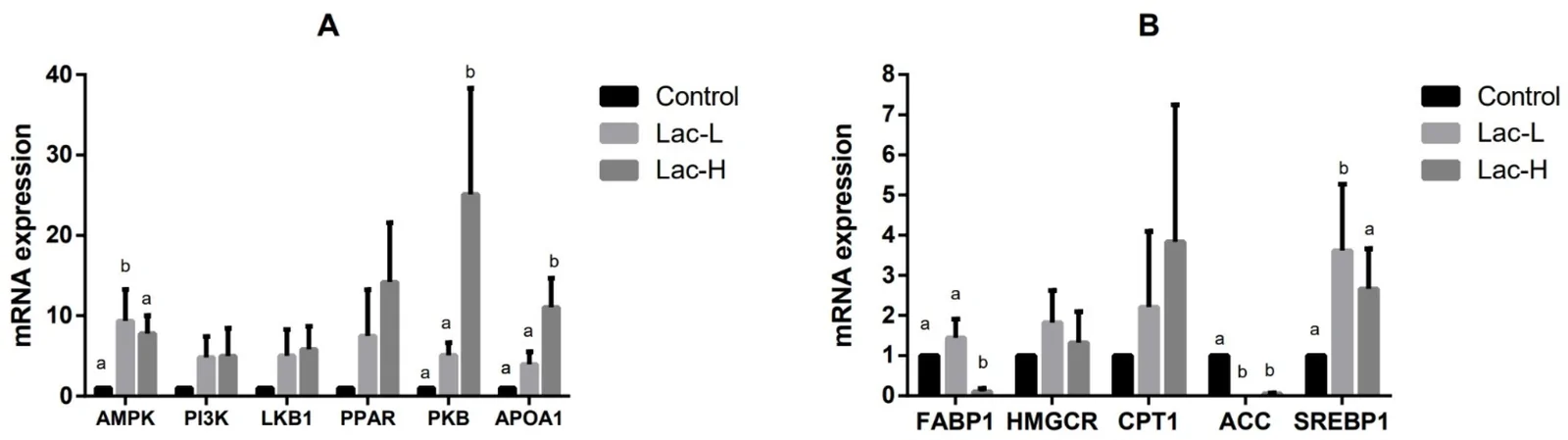
Effects of *Lactococcus* G423 on genes related to the AMPK/PPARα signaling pathway in the liver of AA broilers. Both (**A**,**B**) show RT-qPCR gene expression data, presented in separate panels for clarity. (1) Relative gene expression was calculated using the 2^−ΔΔCT^ method. The control group was set to a normalized value of 1 (i.e., 2^−ΔΔCT^ = 1). Values for treatment groups represent fold-change relative to the control. (2) Mean values without a common superscript letter are significantly different (*p* < 0.05). Data are presented as mean ± SEM (n = 8).

Compared with the control group, the Lac-L and Lac-H groups significantly reduced the mRNA expression of ACC (*p* < 0.05). The Lac-L groups significantly increased the mRNA expression of AMPK and SREBP1 (*p* < 0.05). The Lac-H groups significantly increased the mRNA expression of APOA1 and PKB (*p* < 0.05) and significantly reduced the mRNA expression of FABP1 (*p* < 0.05). Furthermore, these treatment groups increased the mRNA expression of Phosphoinositide 3-kinase (PI3K), liver kinase B1 (LKB1), Peroxisome proliferator-activated receptor (PPAR), HMG-CoA reductase (HMGCR) and Carnitine palmitoyltransferase 1 (CPT1), although these increases were not statistically significant (*p* > 0.05) (Figure 1).

### 3.4. Intestinal Microflora

**Figure 2:**
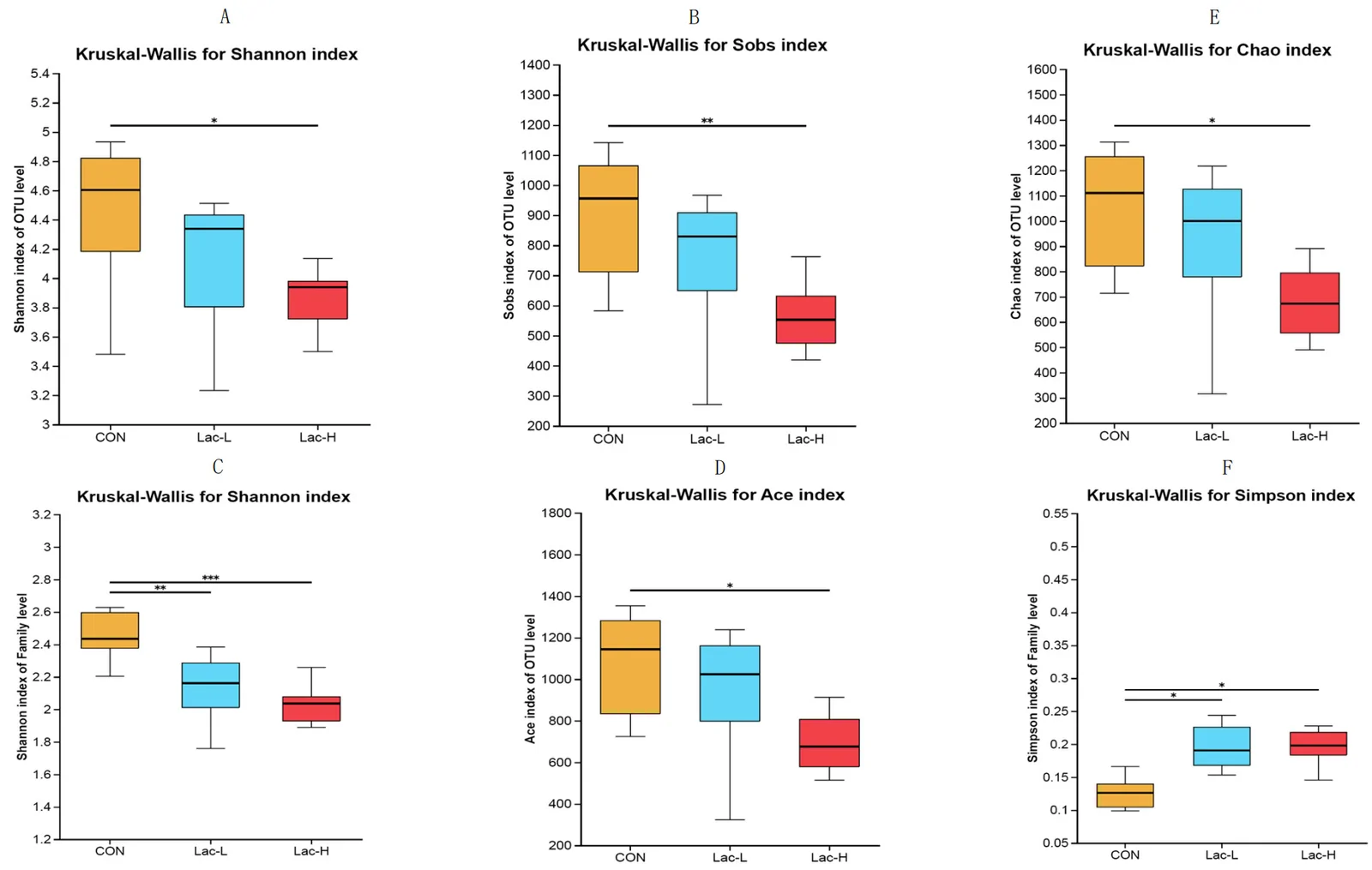
Alpha diversity of intestinal microbiota based on OTU level (n = 8). (**A**,**C**) Shannon indices of cecal microbiota in different groups. (**B**) Sobs index of cecal microbiota in different groups. (**D**) ACE index of cecal microbiota in different groups. (**E**) Chao1 index of cecal microbiota in different groups. (**F**) Simpson index of cecal microbiota in different groups. * represents *p* < 0.05, ** represents *p* < 0.01 and *** represents *p* < 0.001.

**Figure 3:**
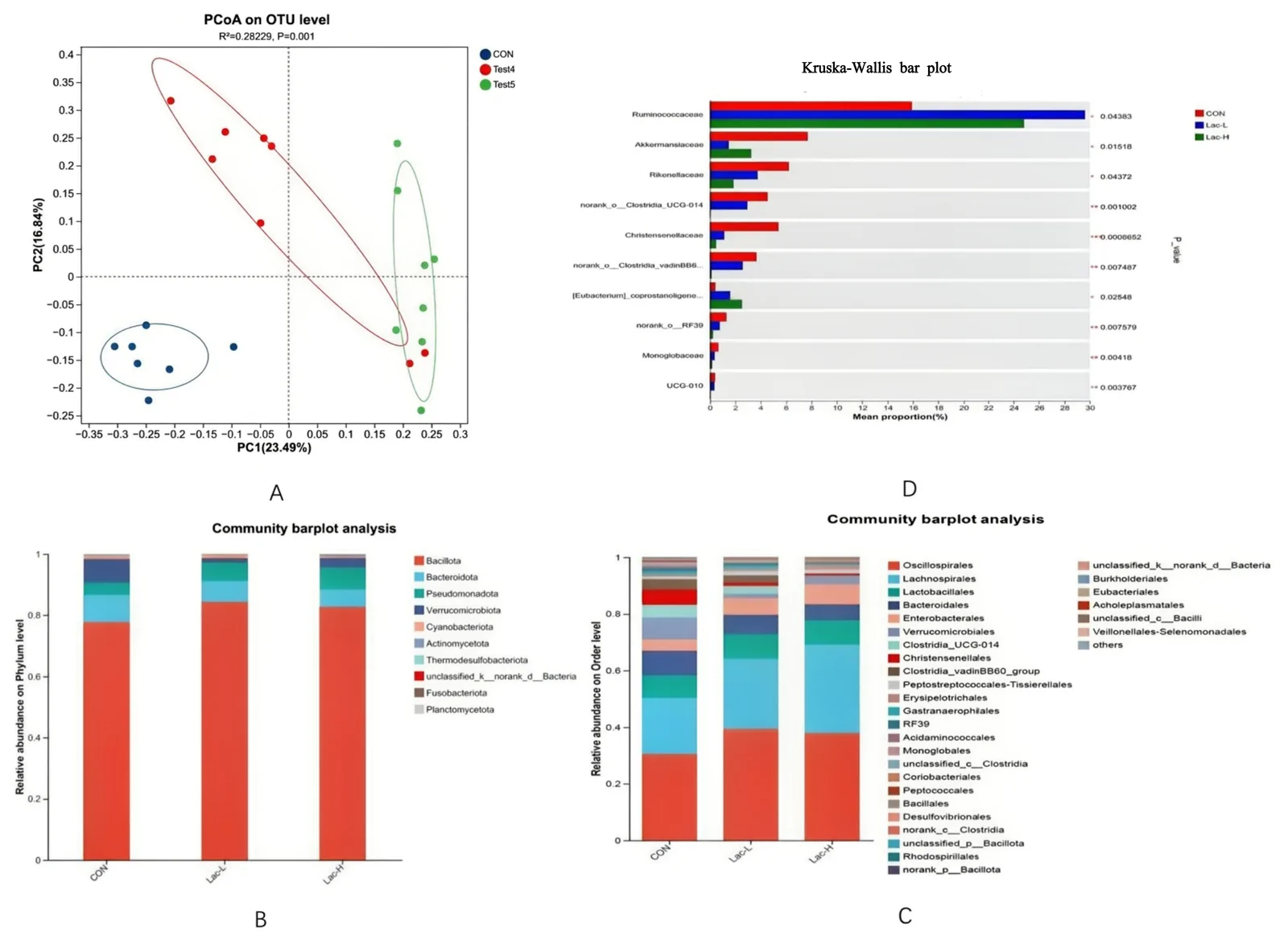
Effects of *Lactococcus* on the gut microbiota of broilers (n = 8). (**A**) Principal coordinate analysis (PCoA) based on weighted UniFrac distance. (**B**) Community composition at the phylum level. (**C**) Community composition at the order level. (**D**) KEGG pathway analysis. * represents *p* < 0.05, ** represents *p* < 0.01 and *** represents *p* < 0.001.

**Figure 4:**
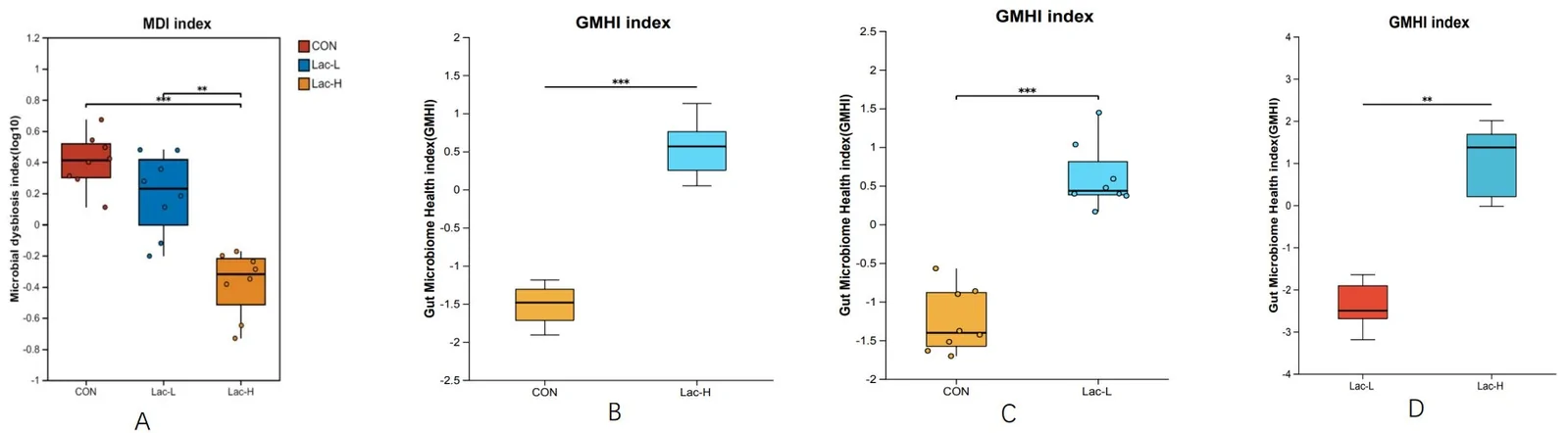
Comparison of GMHI and MDI among the CON, Lac-L, and Lac-H groups. (**A**) MDI among the CON, Lac-L, and Lac-H groups. (**B**) GMHI between the CON and Lac-H groups. (**C**) GMHI between the CON and Lac-L groups. (**D**) GMHI between the Lac-L and Lac-H groups. ** represents *p* < 0.01 and *** represents *p* < 0.001.

To characterize the intestinal microbiota composition of broilers in the three groups, 16S rRNA gene sequencing was performed. A total of 3,854,585 high-quality sequences were obtained. The average Good’s coverage for all samples exceeded 99%, indicating that most microbial species were captured and that sequencing depth was sufficient for robust analysis. As shown in Figure 2, alpha diversity analysis of the gut microbiota revealed that, compared with the CON group, the Chao, Sobs, Shannon, and Ace indices at the OTU level were significantly increased in the Lac_H group (*p* < 0.05). At the family level, the Shannon and Simpson indices were significantly increased in both the Lac_H and Lac_L groups (*p* < 0.05). The effects of *Lactococcus* on the diversity and richness of the intestinal microbiota were evaluated based on these alpha diversity indices. Principal coordinates analysis (PCoA) based on OTU abundance showed that samples from the Lac_H and Lac_L groups were distributed toward the right side of the plot, indicating a substantial shift in microbial structure compared with the CON group. Samples from the CON group clustered separately on the left side, demonstrating that *Lactococcus* G423 altered the structure of the gut microbiota (Figure 3A). At the phylum level, *Bacillota*, *Bacteroidota*, *Pseudomonadota*, and *Verrucomicrobiota* were the most abundant taxa across all samples. The combined relative abundance of these four phyla accounted for 98.4%, 98.7%, and 98.8% in the CON, Lac_L, and Lac_H groups, respectively. The *Bacillota/Bacteroidota* ratio was higher in the Lac_L and Lac_H groups than in the CON group (Figure 3B). At the order level, compared with the CON group, the relative abundance of *Oscillospirales*, *Lachnospirales* and *Lactobacillales* was significantly increased in the Lac_L and Lac_H groups (Figure 3C). Subsequent linear discriminant-analysis effect size (LEfSe) identified significant differences in the abundances of *Ruminococcaceae*, *Akkermansiaceae*, *Rikenellaceae*, *Christensenellaceae*, and *Monoglobaceae* in the Lac_L and Lac_H groups compared with the CON group (*p* < 0.05) (Figure 3D). Compared with the CON group, MDI and GMHI showed significant differences in the Lac_L and Lac_H groups (*p* < 0.05) (Figure 4).

## 4. Discussion

### 4.1. The Effects of Lactococcus G423 on the Growth Performance and Slaughter Performance of AA Broilers

Studies have confirmed that probiotic supplementation can improve nutrient digestion and absorption, enhance animal growth, and improve meat quality [30]. Our study shows that no significant differences (*p* > 0.05) were observed in ADFI, ADG, FCR, breast muscle yield, thigh muscle yield, or abdominal fat percentage between the Lac-L or Lac-H groups and the CON group in AA broilers. Multiple factors influence animal growth performance, including breed, feed nutrition, gender, and age. For example, dietary supplementation with *Bacillus coagulans* and *Lactobacillus plantarum* significantly improved the average daily gain and feed conversion ratio of broilers from days 1 to 42 under lipopolysaccharide stress [31]. Supplementation of broiler feed with *Lactococcus lactis* subsp. BIONCL17752 and varying levels of *fructooligosaccharides* increased broiler body weight by 152, to 171 g per bird [32]. Purnamasari L et al. [33] reported that probiotic supplementation significantly improved average body weight gain, feed efficiency, and carcass yield while reducing abdominal fat, compared with the control group. Fajardo et al. [34] observed no significant effect on final body weight, feed intake, or feed conversion ratio in commercial Ross 308 broilers from days 1 to 42 when fed different species of LAB. Brzoska et al. [35] found that dietary supplementation with *Lactococcus lactis* 847 had no significant effect on body weight, feed conversion ratio, or carcass fat content of broilers, at 42 days of age. The same study reported that *Lactococcus lactis* 847 had no effect on the quantity and proportion of saleable cuts, breast muscle composition, or basic blood parameters [35]. Dankowiakowska A et al. [36] also noted that prebiotics and synbiotics had no influence on carcass yield or pectoral muscle yield. Adding probiotics in related studies can affect production performance, but adding *Lactobacillus* in related studies did not have a significant effect, which is consistent with our experimental conclusions [37]. This result may also be due to specific factors such as the genus and species of the bacteria, the dosage added, and the type of broiler [37,38]. However, a downward trend in FCR and abdominal fat percentage was noted.

### 4.2. The Effect of Lactococcus G423 on Lipid Metabolism in AA Broilers

Lipid metabolism encompasses the synthesis and degradation of fatty acids, triglycerides, and cholesterol. When energy intake exceeds expenditure, the resulting energy surplus promotes triglyceride synthesis. These triglycerides subsequently accumulate in the liver, potentially triggering lipid metabolism disorders characterized by elevated CHO and LDL, alongside reduced HDL levels [37]. Our study shows that both the Lac-L and Lac-H groups significantly reduced serum CHO (*p* < 0.05) and increased HDL levels (*p* < 0.05), compared with the CON group. The ability of probiotics to reduce total cholesterol and triglycerides is closely linked to their bile salt hydrolase activity [39]. After bile is synthesized, it can enter the duodenum to emulsify and dissolve lipids [40]. And bile salts are actively absorbed into the end of the intestine and then return to the liver to form the enterohepatic circulation [41]. Our previous study found that bile salt hydrolase can be detected in multiple bacterial genera, and the study found that *Lactococcus* G423 has a certain regulatory effect on bile acid metabolism by upregulating the levels of 6-hydroxy-5-methoxyindole glucuronic acid [28]. Dietary supplementation with *Lactobacillus* in broiler feed showed no significant effects on serum cholesterol, triglycerides, or total lipids, although a consistent downward trend was observed, suggesting potential lipid-lowering capacity [42]. Another study demonstrated that dietary supplementation with varying concentrations of *Lactococcus lactis* ssp. significantly reduced blood cholesterol and LDL levels [43]. Ding et al. [44] found that dietary supplementation with *Lactobacillus* reduced the content of total CHO and TG in ileal epithelial cells, thereby participating in the lipid metabolism of broilers. The above research analysis suggests that the addition of *Lactococcus* G423 may stimulate the body to use serum cholesterol to synthesize bile, promoting fat emulsification metabolism through the enterohepatic circulation. These findings are consistent with the results of the studies cited above.

### 4.3. The Effect of Lactococcus G423 on the AMPK Signaling Pathway in AA Broilers

Liver lipid metabolism is a complex process associated with alterations in the expression of fat production and lipolytic genes. AMPK, PPAR and CPT1 play important roles in liver fatty acid β-oxidation, whereas ACC, SREBP1, FABP1 and APOA1are key genes controlling fatty acid synthesis and transport [45,46,47,48,49]. The studies indicate that AMPK activation and changing intestinal bacterial communities promote lipid metabolism [50]. For example, *Lactococcus lactis* MQ1-1 and *Qula-derived Limos ilactobacillus fermentum TD-3* activated the AMPK signaling pathway by regulating the gut microbiota and metabolites [51]. The study on the effects of probiotics on AMPK signaling found that administration of three distinct probiotic strains to mice on a high-fat diet activated AMPK, reduced hepatic TG content, and alleviated steatosis [52]. Similarly, *Lactobacillus plantarum* NA136 was reported to inhibit adipose tissue proliferation and promote fatty acid oxidation, thereby improving lipid metabolism through activation of the AMPK-ACC-SREBP1-FAS signaling pathway [5]. In sheep, probiotic supplementation can regulate the ACC, CPT and SREBP by inhibiting the AMPK, resulting in enhanced IMF synthesis and reduced oxidation of fatty acids [53]. The plant-derived lactic acid bacterium *Lactobacillus* ZDY2013 can reduce hepatic fat deposition and promote lipid metabolism by modulating the PI3K/AKT(PKB) signaling pathway [46]. Studies have shown that *Lactobacillus sakei* MJM60958 significantly reduces SREBP1 expression, thereby decreasing lipid accumulation in hepatocytes [54]. Wang et al. [55] found that *Lactobacillus plantarum* 16 significantly upregulated FABP1 expression in the ileal mucosa of broilers, reduced the abundance of harmful intestinal bacteria, and altered intestinal villus morphology. These changes subsequently promoted carbohydrate metabolism and enhanced the growth performance of broilers. *Akkermansia muciniphila* can ameliorate olanzapine-induced hepatic steatosis via the gut microbiota–IGFBP2/APOA1–liver axis. [56] Studies have shown that the expression of certain FABP1 genes is correlated with abdominal fat content in broiler chickens [57].

Specific LAB strains have been directly linked to the hepatic AMPK pathway. For instance, *Lactobacillus johnsonii* 3-1 and *Lactobacillus crispatus* 7-4 reduce ileal TG and TC content, which is associated with upregulation of the hepatic AMPKα/PPARα/CPT-1 pathway and subsequent reduction in abdominal fat deposition in broilers [45]. Beyond direct microbial effects, microbial metabolites, particularly short-chain fatty acids (SCFAs) like acetate, also play a crucial role. Acetate has been shown to improve hepatic glucose and lipid metabolism and reduce ketone body (BHB) production by regulating key signaling pathways, including PPAR, AMPK, and PI3K [58]. For example, in dairy cows, hindgut microbiota such as *Faecalibacterium* and *Methanosphaera* regulate host metabolism precisely, through acetate production [58]. Therefore, we hypothesize that *Lactococcus G423* may similarly activate the hepatic AMPK signaling pathway. This activation could be achieved by modulating the gut microbiota composition and increasing the production of SCFAs, ultimately leading to reduced serum total cholesterol and triglyceride levels. The results of this study demonstrated that, compared with the CON group, the Lac-L and Lac-H groups significantly reduced the mRNA expression of ACC (*p* < 0.05). The Lac-L groups significantly increased the mRNA expression of AMPK and SREBP1 (*p* < 0.05). The Lac-H groups significantly increased the mRNA expression of APOA1 and PKB (*p* < 0.05) and significantly reduced the mRNA expression of FABP1 (*p* < 0.05). These changes possibly subsequently activated the AMPK signaling pathway, which in turn likely exerted regulatory effects on lipid metabolism. Meanwhile, considering the HDL and CHO in this experiment, our findings also showed that *Lactococcus G423* appears to regulate lipid metabolism through the AMPK signaling pathway. A limitation of this study is that the stability of β-Actin was not fully validated under probiotic intervention, which may introduce potential bias in the qPCR quantification.

### 4.4. The Influence of Lactococcus G423 on the Intestinal Microflora Flora of AA Broilers

The gut microbiota regulates hepatic cholesterol utilization and modulates lipid metabolism [59]. The gut microbiota, a complex microbial community in the gastrointestinal tract, is essential for host digestion, metabolism, and immune regulation, with a pronounced role in liver diseases via the gut–liver axis [60]. It can inhibit fat production by influencing bile acids, affecting related receptors, and stimulating associated metabolic pathways [61,62]. The liver and intestine collectively regulate lipid metabolism through this enterohepatic circulation [63,64,65]. Primary bile acids can also enhance gut microbiota abundance, promote the growth of beneficial bacteria, and stimulate the production of short-chain fatty acids (SCFAs) by certain microbial populations [66]. Microbial communities collectively form a highly complex micro-ecosystem that performs functions such as generating SCFAs and promoting lipid metabolism. Key microbes, including *Bacteroides* and *Akkermansia muciniphila*, are noted for their metabolite-mediated effects on hepatic lipid and energy metabolism [67]. Dietary supplementation with compound probiotics in broilers significantly reduced the Shannon and Chao1, indices while decreasing the relative abundance of harmful microorganisms [68]. Moreover, D-mannose has been shown to enhance microbial homeostasis, as reflected by an elevated GMHI and a reduced MDI [69]. In the present experiment, supplementation with *Lactococcus* G423 improved gut microbial balance and enhanced the gut microbiome health index. This was evidenced by significant reductions in the Shannon, Chao1, MDI, and GMHI indices, which align with the findings cited above. Within the gut microbiota, a decrease in the abundance of *Lachnospirales* and *Ruminococcaceae* may reduce the production of certain SCFAs involved in fatty acid metabolism, potentially increasing obesity risk [70]. Conversely, an increase in *Lachnospirales* can modulate lipid metabolism [71]. In this experiment, compared with the CON group, the Lac_L and Lac_H groups showed a significant increase in the abundance of *Lachnospirales*, *Ruminococcaceae*, *Oscillospirales* and *Lactobacillales*, families associated with obesity reduction. The poultry gastrointestinal tract harbors a diverse microbiota, with *Firmicutes* (*Bacillota*), *Bacteroidetes* (*Bacteroidota*), and *Proteobacteria* (*Pseudomonadota*) identified as the most predominant phyla [72]. For instance, supplementation with *Lactobacillus johnsonii* BS15 in broilers increased the populations of *Bacteroides* and *Lactobacillus*, while reducing the abundance of *Enterobacteriaceae* and the *Firmicutes/Bacteroidetes* ratio, ultimately decreasing fat deposition [71]. A lower *Firmicutes/Bacteroidetes* ratio is generally associated with reduced obesity, whereas an increased ratio has been linked to promoted growth in broilers [73]. In the present study, compared with the CON group, the Lac_L and Lac_H groups exhibited an increased *Bacillota/Bacteroidota* ratio. Spearman correlation analysis has indicated that the abundance of *Akkermansiaceae* and *Bacteroidaceae* is positively associated with increased serum cholesterol and LDL levels [74]. Conversely, short-chain fatty acid (SCFA)-producing bacteria, including *Ruminococcaceae* and *Lachnospirales*, were enriched in high-fat diet-fed mice with low cholesterol and triglycerides [75]. *Akkermansia*, as a beneficial microbe in the intestine, is significantly decreased in obesity and type 2 diabetes [76]. There is a strong correlation between liver aspartate levels and the p-AMPK/AMPK ratio, as well as between *Akkermansia* abundance and liver aspartate concentration [77]. *Akkermansia* colonization is related to amino acid metabolism, especially aspartic acid. Aspartic acid can inhibit lipid synthesis by enhancing mitochondrial function through activating the AMPK pathway and promote liver energy balance through the gut–liver amino acid axis [78]. In this experiment, LEfSe revealed that, compared with the CON group, the Lac_L and Lac_H groups also showed significant differences (*p* < 0.05) in the abundances of *Ruminococcaceae*, *Akkermansiaceae*, and *Rikenellaceae*.

*Lactococcus G423* promotes the activation of the hepatic AMPK signaling pathway by modulating the gut microbiota via the gut–liver axis (or enterohepatic circulation). Subsequent activation of this pathway inhibits lipogenesis and upregulates the expression of genes associated with fatty acid oxidation. While we were unable to quantitatively differentiate the proportion of colonized versus excreted strains, the observed alterations in community structure and metabolic pathway enrichment suggest that the supplemented *Lactococcus G423* exerted a significant biological impact on the gut ecosystem, even when functioning solely as a transient passenger.

## 5. Conclusions

The results indicate that dietary supplementation with *Lactococcus* G423 appears to regulate broiler lipid metabolism by modulating the gut microbiota and AMPK signaling pathway. This finding offers new insights into the potential of *Lactococcus* G423, and future studies will focus on its use in livestock production.

## Figures and Tables

**Table 1:** Calculated composition of basal diets and nutrient level.

	Air-Dry Basis	
Composition	1–21 Days	21–42 Days
Ingredients		
Corn	57.50	62.22
Soybean meal	30.50	29.00
Corn gluten meal	5.00	1.00
Soybean oil	1.50	6.00
Sodium chloride	0.30	0.30
Dicalcium phosphate	1.65	1.70
Limestone	0.70	0.80
Methionine	0.35	0.25
Choline	0.15	0.15
Multivitamin premix ^a^	0.03	0.03
Mineral premix ^b^	0.10	0.10
Total	100	100
Nutrient level		
Metabolizable energy (MJ/kg)	12.33	12.50
Crude protein (%)	22.75	21.72
Lysine (%)	1.41	1.22
Methionine + Cysteine (%)	0.91	0.86
Ca (%)	1.07	0.60
Total P (%)	0.70	0.68
Available P (%)	0.46	0.45

^a^: Content per kilogram of diet: 12,500 IU of vitamin A, 4500 IU of vitamin D3, 40 IU of vitamin E, 5 mg of vitamin K, 5 mg of vitamin B1, 9 mg of vitamin B2, 6 mg of vitamin B6, 0.03 mg of vitamin B12, 0.3 mg of biotin, 0.6 mg of folic acid, 60 mg of niacin, and 20 mg of pantothenic acid. ^b^: Content: 10 mg of Cu (CuSO_4_·5H_2_O), 1.2 mg of I (KI), 20 mg of Fe (FeSO_4_·7H_2_O), 60 mg of Mn (MnSO_4_·H_2_O), 0.4 mg of Se (NaSeO_3_), and 100 mg of Zn (ZnSO_4_).

**Table 2:** Parameters of primer pairs for the genes.

Gene	Primer Sequence	Product Size	GenBank Accession
*PPARα*	F: 5′-TAACGGAGTTCCAATCGC-3′ R: 5′-AACCCTTACAACCTTCACAA-3′	166 bp	NM 001001464.1
*PKB/AKT*	F: 5′-CTGATGATGCCAAGGAGATT-3′ R: 5′-TGGTCAGGAGGAGTGATTGT-3′	109 bp	NM 205055
*AMPK*	F: 5′-AAGGTTGGCAAGCATGAGTT-3′ R: 5′-TTCTGGGCCTGCATATAACC-3′	120 bp	NM 001039603
*PI3K*	F: 5′-CGGATGTTGCCTTACGGTTGT-3′ R: 5′-GTTCTTGTCCTTGAGCCACTGAT-3′	162 bp	NM 001004410.1
*ACC*	F: 5′-CACTTCGAGGCGAAAAACTC-3′ R: 5′-GGAGCAAATCCATGACCACT-3′	124 bp	NM 205505.1
*CPT1*	F: 5′-CAATGAGGTACTCCCTGAAA-3′ R: 5′-CATTATTGGTCCACGCCCTC-3′	120 bp	NM 001012898.1
*HMGCR*	F: 5′-AGCTGCAACCCTGAGGAAACT-3′ R: 5′-AGCCATCACTGTAGCACACAC-3′	113 bp	NM_204485.3
*SREBP1*	F: 5′-GAGGAAGGCCATCGAGTCA-3′ R: 5′-GGAAGACAAAGGCACAGAGG-3′	123 bp	NM_204126.3
*LKB1*	F: 5′-GGGGAGACAGAAGGGAACAGA-3′ R:5′-TGAGAGGGATGCTTGAATACGA-3′	130 bp	NM 001045833.1
*APOA1*	F: 5′-CCTTCTGGCAGCACGATGAGC-3′ R: 5′-CAGCGTGTCCAGGTTGTCAGC-3′	170 bp	XM_015297971.2
*FABP1*	F: 5′-TCACTGGAAAGTACGAGC-3′ R: 5′-GCATGCAGGGTCTCTAGATT-3′	219 bp	NM_204192.4
*β-actin*	F: 5′-TGCGTGACATCAAGGAGAAG-3′ R: 5′-TGCCAGGGTACATTGTGGTA-3′	183 bp	NM_205518.1

**Table 3:** Effect of *Lactococcus* G423 on growth performance and carcass characteristics in broilers.

Items	Control	Lac-L	Lac-H	*p*-Value
ADFI	136.41 ± 1.38	133.2 ± 1.41	137.49 ± 2.03	0.093
ADG	59.05 ± 0.833	59.81 ± 0.414	60.83 ± 0.757	0.099
FCR	1.504 ± 0.024	1.467 ± 0.02	1.465 ± 0.017	0.225
Breast muscle (%)	32.4 ± 0.63	30.8 ± 0.52	30.5 ± 0.38	0.054
Thigh muscle (%)	29.42 ± 0.68	30.4 ± 1.00	30.5 ± 0.68	0.105
Abdominal fat (%)	1.48 ± 0.18	1.38 ± 0.23	1.24 ± 0.19	0.435

ADFI = average daily feed intake. ADG = average daily gain. F/G = feed/gain. Values are expressed as means ± SEM, and n = 8 for all groups. One-way analysis of variance (ANOVA) was used to test the overall difference among the three treatment groups, and the *p*-value in the ANOVA table was used for the *p*-value for the overall test; after the overall difference was found to be significant, the LSD method was used for pairwise comparisons between groups, and the values in the table were used for the *p*-values for the pairwise comparisons of each group.

**Table 4:** Effect of *Lactococcus* G423 on serum biochemical parameters in broilers.

Items	Control	Lac-L	Lac-H	*p*-Value
TG (mg/dL)	0.57 ± 0.18	0.54 ± 0.10	0.48 ± 0.47	0.496
LDL (mg/dL)	2.52 ± 0.23	2.16 ± 0.31	2.09 ± 0.25	0.468
HDL (mg/dL)	2.03 ± 0.13 ^a^	2.52 ± 0.16 ^ab^	2.86 ± 0.23 ^b^	0.017
CHO (mg/dL)	3.59 ± 0.35 ^a^	2.70 ± 0.25 ^b^	2.52 ± 0.16 ^b^	0.020

TG, triglyceride; HDL, high-density lipoprotein; LDL, low-density lipoprotein; CHO, cholesterol. Values are expressed as means ± SEM, and n = 8 for all groups. One-way analysis of variance (ANOVA) was used to test the overall difference among the three treatment groups, and the *p*-value in the ANOVA table was used for the *p*-value for the overall test; after the overall difference was found to be significant, the LSD method was used for pairwise comparisons between groups, and the values in the table were used for the *p*-values for the pairwise comparisons of each group. Different letters in the same row indicate a significant difference between the two groups (*p* < 0.05).

## Data Availability

The data presented in the study are deposited in the figshare repository, accession number https://doi.org/10.6084/m9.figshare.32030649.
